# Anti-Obesity Effects of Combined *Cornus officinalis* and *Ribes fasciculatum* Extract in High-Fat Diet-Induced Obese Male Mice

**DOI:** 10.3390/ani11113187

**Published:** 2021-11-08

**Authors:** Eunkuk Park, Chang-Gun Lee, Hyoju Jeon, Hyesoo Jeong, Subin Yeo, Yoonjoong Yong, Seon-Yong Jeong

**Affiliations:** 1 Department of Medical Genetics, Ajou University School of Medicine, Suwon 16499, Korea; jude0815@hotmail.com (E.P.); dangsunsang@naver.com (C.-G.L.); wjsgywn0315@ajou.ac.kr (H.J.); 2 Department of Biomedical Sciences, Ajou University Graduate School of Medicine, Suwon 16499, Korea; 3 Nine B Company, Daejeon 34121, Korea; jhyesoo921@gmail.com (H.J.); snsnans@naver.com (S.Y.); yoonjoong9b@gmail.com (Y.Y.)

**Keywords:** anti-obesity, *Cornus officinalis*, *Ribes fasciculatum*, high-fat diet, obese male mice

## Abstract

**Simple Summary:**

Obesity is a general health problem representing a high risk factor for a low-quality lifestyle. Various Food and Drug Administration-approved pharmacological medications have been established for the treatment and prevention of obesity. However, some pharmacotherapies present adverse effects and limited long-term use. Natural herbal medicines as alternative cures have shown low side-effects and suitability for long-term treatment. *Cornus officinalis* and *Ribes fasciculatum* (CR) are well-known oriental plants used for health dietary supplements and herbal medicine. This study examined the anti-obesity effect of CR in high-fat diet (HFD)-induced obese male mice. Treatment of CR extract prevented body weight gain through the downregulation of adipogenic inducible genes and recovered the dysregulated energy metabolism in HFD-induced obese male mice. Therefore, CR reduced elevated biochemical obesity parameters in plasma, as well as inhibited hepatic steatosis in the liver and adipocyte size increase in fat tissue. These findings of the study reveal the potential anti-obesity effects of CR as an herbal medicine.

**Abstract:**

Medicinal plants are widely used as supplements for the treatment of various diseases because of their few side-effects. Here, we examined the anti-obesity effects of a mixture extract of *Cornus officinalis* and *Ribes fasciculatum* (CR) in high-fat diet (HFD)-induced obese male mice. Four week old male C57BL/6J mice were fed a normal diet (ND) or 60% high-fat diet (HFD) with different concentrations of CR extracts (75, 150, and 300 mg/kg/day) by oral administration for 12 weeks. CR extract administration prevented HFD-induced weight gain, hepatic steatosis, and adipocyte enlargement through the downregulation of adipogenesis-associated genes in obese male mice. In addition, CR administration improved the impaired glucose metabolism, insulin action, biochemical obesity parameters, and metabolic profiles in HFD-induced male mice. Consequently, the CR extract exhibited beneficial effects on HFD-induced systemic metabolic challenges. Taken together, our findings suggest that CR extract may be a potent therapeutic supplement for the treatment and prevention of obesity.

## 1. Introduction

Obesity is a complex medical condition involving abnormal body fat accumulation and associated health problems. Obesity is caused by genetics, endocrine disorders, energy imbalances (such as excessive uptake of calories), genetic syndromes, hormonal changes, and socioeconomic and mental factors [1,2]. Excessive weight gain leads to an unnatural body fat distribution, resulting in an increased risk of obesity-related complications, including high blood pressure, type 2 diabetes, atherosclerosis, and cancers [3,4]. Healthy lifestyle changes such as improvements in eating behaviors, physical activity, and appropriate levels of sleep are recommended as effective approaches to prevent obesity in humans [5].

As behavioral strategies have limited success for some people, there are Food and Drug Administration (FDA)-approved pharmacological medicines that can decrease appetite and block fat absorption through control of the brain and gastrointestinal tract [6]. Although these medicines combined with healthy lifestyle changes can help weight loss, some pharmacotherapies have side-effects and limitations for long-term use.

Oriental herbal medicines have been broadly utilized as alternative modern therapies for the treatment of various diseases due to fewer adverse effects and their suitability for long-term treatment [7,8,9]. Several studies have demonstrated that combined herbal medicines have synergistic effects when used as a single treatment, while avoiding adverse effects [10,11]. Recently, a combination of *Schisandra chinensis* and *Ribes fasciculatum* was shown to promote synergistic neuroprotective effects on hydrogen peroxide-induced PC12 neuronal cell death and scopolamine-induced memory impairment in rats [12]. Moreover, mixtures of five herbal plant extracts showed complex protective effects against atopic dermatitis-like skin lesions [13].

*Cornus officinalis* (CO) and *Ribes fasciculatum* (RF) are commercialized natural plants for health supplements and are used as oriental medicines in East Asia. Recent studies have reported that CO ethanolic extract has inhibitory effects on inflammatory responses, oxidative stress, and allergic responses [14] and improves liver function in nonalcoholic fatty liver disease [15]. Similarly, several reports have suggested that RF extract exhibits antiaging effects in *Caenorhabditis elegans* dependent on the insulin/IGF signaling pathway [16] and prevents allergic inflammation in LPS-stimulated macrophage cells [17]. In addition, previous studies have demonstrated that the combination of CO and RF (CR) extract improves estrogenic activity and anti-osteoporotic effects [18], has along with anti-obesity effects in female mice [19] and women [20].

Regarding gender differences in the pharmacological effects on obesity [21,22], the anti-obesity effects of CR extract on male mice have not been reported; it is, therefore, unclear whether the anti-obesity effect of CR is gender-specific. The present study aimed to examine the anti-obesity effects of CR in high-fat diet (HFD)-induced obese male mice.

## 2. Materials and Methods

### 2.1. Preparation of Cornus officinali (CO) and Ribes fasciculatum (RF) Extracts and Their Chemical Composition

*Cornus officinalis* (CO) raw material was obtained from Icheon City and Yangpyeong City (Gyeonggi, Korea), and *Ribes fasciculatum* (RF) plants were obtained from Goesan City (Chungbuk, Korea). Air-dried CO and RF were extracted in 1 L of aqueous ethanol solution in a water bath at 60 °C for 8 h, followed by filtration using qualitative low-ash filter paper (CHMLAB, Terrassa, Barcelona, Spain) to remove debris. The following procedure was followed after the concentration of the sample extracts: ethanolic extracts of CO and RF were evaporated over 25° Brix using a rotary vacuum evaporator at 45 °C; concentrated CO and RF extracts were then dried and stored at −20 °C before use.

HPLC–DAD profiling of major compounds in the CO and RF extracts was performed to obtain qualitative and quantitative information. Morroniside and loganin were detected in the CO extract, and 4-hydroxybenzoic acid (4-HBA) was detected in the RF extract. The identity of these compounds was confirmed by the comparison of retention times of the standard compounds (Supplementary Figure S1). The major compounds responsible for the anti-obesity effect were found to be morroniside, loganin, and 4-HBA. Next, we developed a method for the separation and quantitative analysis of loganin and 4-HBA using HPLC with diode array detection. The following validation characteristics were assessed: specificity (Supplementary Figure S2), linearity (Supplementary Table S1), accuracy (Supplementary Table S2), and precision (Supplementary Table S3). CR extracts were standardized on the basis of the concentrations of loganin and 4-HBA (loganin: 2.2–3.4 mg/g and 4-HBA: 3.1–4.6 mg/g).

### 2.2. Animal Studies

All animal experiments were approved by the Institutional Animal Care and Use Committee (IACUC) of Ajou University School of Medicine (2020-0036) and conducted in accordance with institutional guidelines. Four week old male C57BL/6J mice were purchased from DBL Co., Ltd. (Chungbuk, Korea). Mice were maintained in a specific-pathogen-free facility at the Laboratory Animal Research Center of Ajou University Medical Center, with controlled conditions (temperature (23 ± 2 °C), humidity (55 ± 5%), and illumination (12 h light/dark cycle)) and provided with sterile food and water ad libitum. For the experiment, mice were fed a normal diet (ND) or 60% high-fat diet (HFD) mixed with different concentrations of combined CR and RF in a 7:3 ratio (75, 150, and 300 mg/kg/day, *n* = 5 in each group) by oral administration for 12 weeks. Total body weight was monitored during the experiment using an electronic scale, and mouse tissue and plasma samples were collected at the end of the experiment.

### 2.3. Histological Analysis and Quantification of Adipose Tissue Area

Mouse liver and fat tissues were fixed with 4% paraformaldehyde for 24 h and embedded in paraffin. The samples were sectioned (3 μm) and stained with hematoxylin and eosin (H&E) for histopathological analysis. Representative images were visualized using a light microscope. For quantification of adipocyte area, 100 cells from each slide were evaluated using ImageJ software (NIH, Bethesda, MD, USA).

### 2.4. Oral Glucose Tolerance Test (OGTT) and Insulin Tolerance Test (ITT)

Mice were fasted for 12 h, and glucose was administered by oral gavage (2 g/kg; Sigma-Aldrich, G8270, St. Louis, MO, USA). For the insulin tolerance test (ITT), mice were fasted for 6 h, and insulin was intraperitoneally injected (1 U/kg, Sigma-Aldrich, I0516). Blood samples were obtained at different time periods (0, 15, 30, 45, 60, 90, 120, and 180 min) from the tail vein, and blood glucose levels were measured using a Gluco Dr. Auto Blood Glucose Monitoring System (Allmedicus, Anyang, Korea).

### 2.5. Metabolomic Cage Monitoring System and Analysis of Biochemical Obesity Indicators in Plasma

Metabolic cages accommodating 12 mice (TSE PhenoMaster, TSE systems, Bad Homburg, Germany) were used to measure the O_2_ consumption (VO_2_), CO_2_ production (VCO_2_), and locomotor activity. Food intake and water consumption were individually measured. The respiratory exchange ratio (RER) and energy expenditure (EE) were calculated. At the end of the experiment, blood samples were obtained, and plasma levels of AST, ALT, TG, and total cholesterol were examined using DRI-CHEM NX500 (Fujifilm global, Minato-ku, Tokyo, Japan).

### 2.6. Quantiative Reverse-Transcription Polymerase Chain Reaction (qRT-PCR)

To examine adipogenesis-associated genes, total RNA was isolated from abdominal adipose tissue using TRIzol reagent (Invitrogen, Carlsbad, CA, USA) according to the manufacturer’s instructions. Complementary DNA (cDNA) was synthesized using a cDNA synthesis kit (RevertAid™ H Minus First Strand cDNA Synthesis Kit (Fermentas, Hanover, NH, USA). qRT-PCR was performed using a SYBR Green I qPCR kit (TaKaRa, Shiga, Japan), in accordance with the manufacturer’s recommendations. Gene-specific primers for adipogenesis-associated genes in adipose tissues were as follows: forward 5′–GCG GGA ACG CAA CAA CAT C–3′ and reverse 5′–GTC ACT GGT CAA CTC CAG CAC–3′ for mouse *Cebpα*, forward 5′–AAG GTG AAG AGC ATC ATA ACC CT–3′ and reverse 5′–TCA CGC CTT TCA TAA CAC ATT CC–3′ for mouse *Fabp4*, forward 5′–GGA AGA CCA CTC GCA TTC CTT–3′ and reverse 5′–GTA ATC AGC AAC CAT TGG GTC–3′ for mouse *Pparg*, forward 5′–AAG ATG TAC CCG TCC GTG TC–3′ and reverse 5′–TGA AGG CAG GCT CGA GTA AC–3′ for mouse *Srebp1*, forward 5′–GGA GGT GGT GAT AGC CGG TAT–3′ and reverse 5′–TGG GTA ATC CAT AGA GCC CAG–3′ for mouse *Fasn*, forward 5′–GAC CTT GTG TCC TCC GCT TAT–3′ and reverse 5′–GAC CTT GTG TCC TCC GCT TAT–3′ for mouse *Plin2*, forward 5′–ACA CTG GTC CTA GCT GTA TTC T–3′ and reverse 5′–CCA GCC ACG TTG CAT TGT A–3′ for mouse *Slc2a4*, and forward 5′–TGA CCA CAG TCC ATG CCA TC–3′ and reverse 5′–GAC GGA CAC ATT GGG GGT AG–3′ for mouse *Gapdh*. Relative expression levels were normalized to mouse *Gapdh* expression, and the expression levels of each group were expressed as fold changes compared to the HFD group. Fold change was presented as 2^−ΔΔCt^ (ΔΔCt = ΔCt_HFD group_ − ΔCt_experiment group_).

### 2.7. Statistical Analysis

The results are presented as the mean ± standard error of the mean (SEM), and statistical analyses were conducted using GraphPad Prism 9.0 (GraphPad, San Diego, CA, USA). Comparisons between two groups were evaluated by unpaired *t*-tests, and multiple comparisons between groups were determined by one-way analysis of variance (ANOVA), followed by Tukey’s honestly significant difference (HSD) post hoc test. A probability value less than 0.05 (*p* < 0.05) was considered to be statistically significant.

## 3. Results and Discussion

### 3.1. Anti-Obesity Effects of CR in High-Fat Diet (HFD)-Induced Obese Male Mouse

Epidemiological studies have demonstrated that a high-fat diet (HFD) increases the risk of obesity development, and there is a positive relationship between the amount of dietary fat intake and the degree of obesity [23,24]. In humans, many studies have demonstrated that gender differences are observed in energy consumption, requirements for fat distribution, and metabolism [25]. Previous studies revealed that CR administration attenuated obesity development in OVX mice [18], HFD-induced female mice [19], and women [20]. Despite the higher prevalence of obesity in women than in men, this study selected HFD-induced obese male mice as an obese animal model as there is a paucity of information on CR effectiveness in males.

We examined the anti-obesity effects of CR extract in HFD-induced obese male mice. Thirty C57BL/6J (four week old male mice) were fed a 60% high-fat diet to induce weight gain and divided into six groups: (1) normal diet (ND); (2) 60% high-fat diet only (HFD); (3) 60% high-fat diet + CR 75 mg/kg (HFD + CR75); (4) 60% high-fat diet + CR 150 mg/kg (HFD + CR150); (5) 60% high-fat diet + CR 300 mg/kg (HFD + CR300); (6) normal diet + CR 300 mg/kg (ND + CR300). The combination of CR and RF extract in a 7:3 ratio was mixed with either normal or 60% high-fat calories and provided for 12 weeks. On the last day of the animal experiment, body weight, oral glucose tolerance test (OGTT), and insulin tolerance test (ITT) were analyzed, and histological assessments of liver tissues and abdominal fat were visualized using hematoxylin and eosin (H&E) staining. During administration, food intake did not differ between the groups. In vivo toxic effects were investigated by comparing the normal diet and normal diet treated with CR 300 mg/kg for 12 weeks, but body weight, OGTT, and ITT did not differ between the two groups (Supplementary Figures S3–S5).

**Figure 1:**
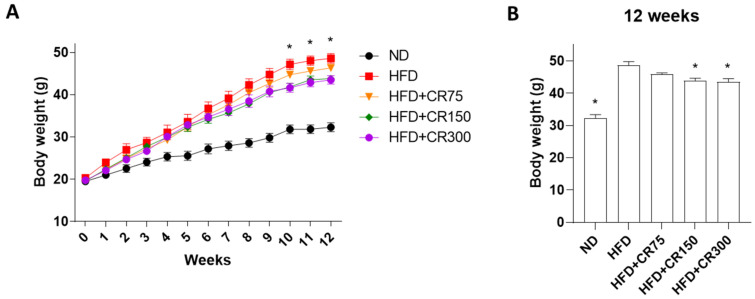
Anti-obesity effects of CR extract in HFD-induced obese male mice. Mice were given normal diet or high-fat diet with coadministration of CR extract at different concentrations (75, 150, and 300 mg/kg/day, *n* = 5 for each group). (**A**) Body weight changes for mice during 12 weeks of the experiment; * *p* < 0.05 vs. HFD + CR300. (**B**) Total mouse body weight at the end of the experiment. ND, normal diet; HFD, high-fat diet; CR, CR extract administration; * *p* < 0.05 vs. HFD (one-way ANOVA with Tukey’s honestly significant difference post hoc test).

HFD-induced obese male mice showed increased body weight compared to the normal diet group (Figure 1A,B). Treatment with CR extract at 150 and 300 mg/kg/day in high-fat diet-fed male mice significantly prevented body weight gain after 10 weeks of administration, compared to the HFD group (Figure 1A,B). These results suggest that the combination of CR extract inhibited weight gain in HFD-induced obese male mice.

### 3.2. Oral Glucose Tolerance Test (OGTT) and Insulin Tolerance Test (ITT) of High-Fat Diet (HFD)-Induced Obese Male Mice

Obesity is a critical risk factor in the pathogenesis of type 2 diabetes, characterized by impaired glucose metabolism and insulin resistance [26,27]. Insulin and glucagon play a principal role in the regulation of nutritional balance and cellular energy supply, leading to the maintenance of appropriate systemic glucose concentrations in the blood [28]. Glucose is the most important stimulator of insulin secretion that activates the anabolic progression of the fed state, resulting in the initiation of glycolysis and fatty-acid synthesis [29].

Oral glucose tolerance tests (OGTTs) are widely used in clinical practice and physiological tests to evaluate normal or impaired glucose metabolism [30]. In addition, the insulin tolerance test (ITT) is used to investigate insulin action in the whole body by measuring blood glucose levels in response to insulin [31]. The HFD-induced obese animal model is well characterized by precipitous body weight gain with increased energy intake and consequently decreased metabolic efficiency with insulin resistance [32]. The present study used C57BL/6J male mice as an HFD-induced obesity model that showed impaired glucose-mediated insulin secretion to investigate obesity related to insulin resistance. Generally, glucose tolerance in a healthy mouse is characterized by a rapid growth in blood glucose, while insulin tolerance shows decreased blood glucose levels.

**Figure 2:**
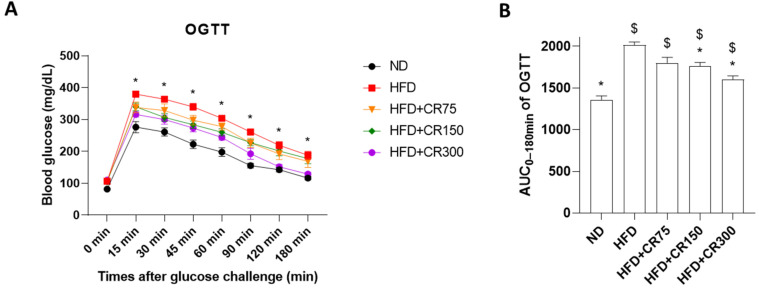
Oral glucose tolerance test (OGTT) for high-fat diet (HFD)-induced obese male mice given diets with different concentrations of CR extract (75, 150, and 300 mg/kg/day, *n* = 5 for each group) for 12 weeks. (**A**) Time course of blood glucose levels during the total glucose tolerance test; * *p* < 0.05 vs. HFD + CR300. (**B**) AUC_0–180min_ values of OGTT were calculated. Data are presented as the mean ± SEM (*n* = 5 for each group). ND, normal diet; HFD, high-fat diet; CR, CR extract administration; * *p* < 0.05 vs. HFD, ^$^ *p* < 0.05 vs ND (one-way ANOVA with Tukey’s honestly significant difference post hoc test).

**Figure 3:**
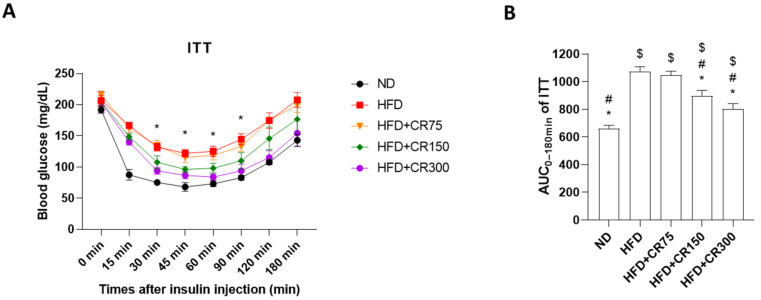
Insulin tolerance test (ITT) for HFD-induced obese mice given different concentrations of CR extract (75, 150, and 300 mg/kg/day, *n* = 5 for each group) for 12 weeks. (**A**) Time course of blood glucose levels during the total insulin tolerance test; * *p* < 0.05 vs. HFD + CR300. (**B**) AUC_0–180min_ values of ITT were calculated. Data are presented as the mean ± SEM (*n* = 5 for each group). ND, normal diet; HFD, high-fat diet; CR, CR extract administration; * *p* < 0.05 vs. HFD, ^#^ *p* < 0.05 vs. HFD + CR75, ^$^ *p* < 0.05 vs. ND (one-way ANOVA with Tukey’s honestly significant difference post hoc test).

During OGTT, the blood glucose concentrations reached their highest levels at 15 min after glucose administration, followed by moderately decreasing glucose levels, reaching basal levels 180 min after the glucose challenge, thus indicating proper glucose metabolism. On the other hand, ITT significantly decreased after insulin administration, followed by recovery of glucose levels within 180 min. Both HFD-induced and CR-administered male mice presented impaired glucose levels during OGTT and ITT, compared to the normal diet group (Figure 2 and Figure 3). However, CR (150 and 300 mg/kg/day)-treated mice showed significant improved glucose tolerance and insulin action, compared to the HFD group (Figure 2 and Figure 3). These results indicate that CR treatment recovered impaired glucose metabolism and insulin resistance in the HFD-induced obesity mouse model.

### 3.3. Effects of CR on Plasma Levels of Biochemical Obesity Indicators in HFD-Induced Obese Male Mice

A positive relationship between fasting and postprandial glucose and increased liver enzymes has been reported [33], and hyper- or hypoglycemic controls stimulated increased alanine aminotransferase (ALT) levels with morphological changes in the liver [34]. In addition, elevated ALT induces the production of triglycerides and total cholesterol [35]. To investigate the effects of CR on plasma levels of lipids and liver enzymes, blood chemistry analyses for aspartate aminotransferase (AST), ALT, triglyceride, and total cholesterol were measured. HFD-fed mice showed increased body weight through elevated glucose levels and decreased glucose uptake, resulting in hyperlipidemia [36].

**Figure 4:**
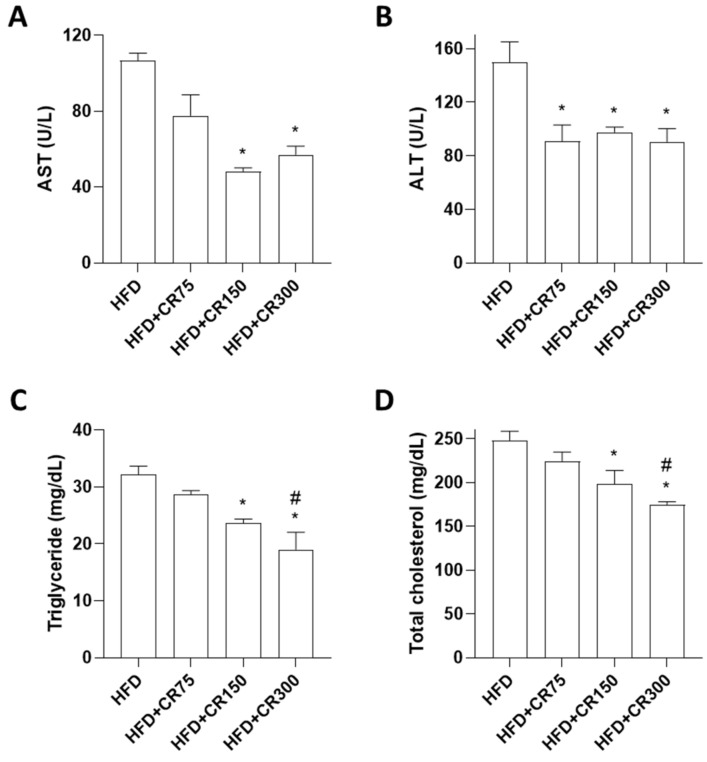
Effects of CR extract on plasma profiles associated with HFD-induced obesity. Plasma levels of (**A**) AST, (**B**) ALT, (**C**) triglyceride, and (**D**) total cholesterol were examined using DRI-CHEM NX500. HFD, high-fat diet; CR, CR extract administration; * *p* < 0.05 vs. HFD; ^#^ *p* < 0.05 vs. HFD + CR75 (one-way ANOVA with Tukey’s honestly significant difference post hoc test).

In line with previous studies, significant increases in AST, ALT, triglyceride, and total cholesterol were observed in HFD-induced obese mice (Supplementary Figure S6). However, mice treated with CR (150 and 300 mg/kg/day) showed significant reduced liver enzymes (AST and ALT) (Figure 4A,B), triglyceride, and total cholesterol (Figure 4C,D), indicating hypocholesterolemic and hypoglycemic activities in HFD-induced obese mice. One study suggested that increased glucose levels enhanced the lipid accumulation in liver and fat tissues [37].

### 3.4. Effects of CR on Adipogenesis in HFD-Induced Obese Male Mice

**Figure 5:**
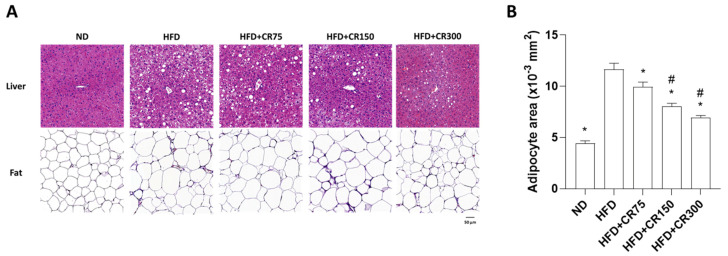
Effects of combined CR extract administration on HFD-induced hepatic steatosis and adipose tissue enlargement. (**A**) Hematoxylin and eosin staining of mouse liver and adipose tissue. (**B**) Adipose tissue area was quantified using ImageJ software. ND, normal diet; HFD, high-fat diet; CR, CR extract administration; * *p* < 0.05 vs. HFD; ^#^ *p* < 0.05 vs. HFD + CR75 (one-way ANOVA with Tukey’s honestly significant difference post hoc test).

We investigated the histological morphology of hematoxylin and eosin (H&E)-stained liver and abdominal visceral fat tissues (Figure 5A). Images in HFD mice showed fatty hepatocyte deposition with a high degree of cytoplasmic vacuoles in the liver and significant adipocyte size enlargement in the fat tissue. However, HFD mice treated with CR at 300 mg/kg/day prevented severe hepatic steatosis and adipocyte increase (Figure 5A,B). These results suggest that CR treatment inhibited fat accumulation in liver and fat tissues via the reduction of AST, ALT, triglyceride, and total cholesterol in HFD-induced obese male mice.

**Figure 6:**
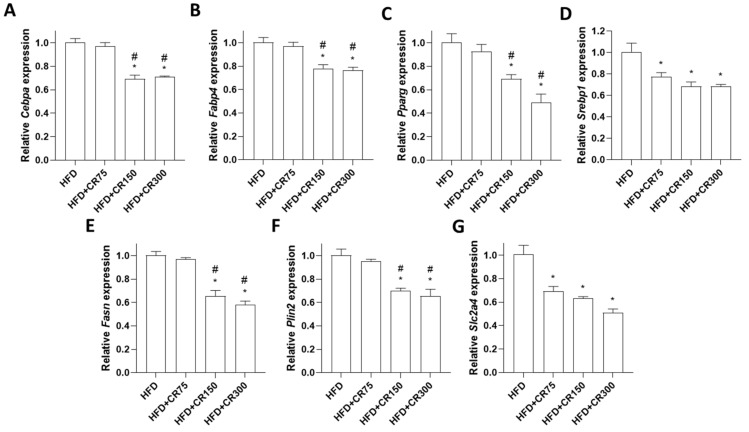
Effects of CR extract administration on the expression of HFD-induced adipogenesis associated genes. mRNA expression levels of adipogenesis-associated genes in adipose tissue were examined by qRT-PCR. Relative gene expression levels of (**A**) *Cebpα*, (**B**) *Fabp4*, (**C**) *Pparγ*, (**D**) *Srebp1*, (**E**) *Fasn*, (**F**) *Plin2*, and (**G**) *Slc2a4* were normalized with mouse *Gapdh* expression. HFD, high-fat diet; CR, CR administration; * *p* < 0.05 vs. HFD; ^#^ *p* < 0.05 vs. HFD + CR75 (one-way ANOVA with Tukey’s honestly significant difference post hoc test).

To further examine the specific adipogenic effects of CR extract, mRNA expression of adipogenesis-associated transcription factors in adipose tissue was analyzed by quantitative reverse transcription PCR (qRT-PCR). Previously, CR administration decreased the expression of adipogenic markers such as CCAAT/enhancer-binding protein alpha (*Cebpα*), perilipin1, fatty acid-binding protein 4 (*Fabp4*), adiponectin, peroxisome proliferator-activated receptor gamma *(Pparγ*), and sterol regulatory element-binding protein (*Srebp)* in 3T3-L1 preadipocyte cells [18,19] and *Cebpα*, *Fabp4*, *Pparγ*, and *Srebp* in adipose tissue of HFD-induced obese female mice [19]. Consistent with the previous results, mRNA expression of *Cebpα*, *Fabp4*, *Pparγ*, and *Srebp* in the abdominal fat tissues was also inhibited by CR treatment in HFD-induced male mice in the present study (Figure 6A–D). In addition, expression levels of other adipogenic genes, such as fatty-acid synthase (*Fasn*), perilipin 2 (*Plin2*), and solute carrier family 2 member 4 (*Slc2a4*) were evaluated. *Fasn* is a central enzyme that induces lipogenesis in adipose tissue and is associated with metabolic alterations in overnutrition [38]. *Plin2* controls lipid homeostasis, which regulates lipid storage and metabolism in adipose tissue [39]. *Slc2a4* is a glucose transporter that activates triglyceride storage and fatty-acid synthesis [40]. The results of the present study showed that the CR administration group showed decreased mRNA expression of *Fasn*, *Plin2*, and *Slc2a4* in adipose tissue (Figure 6E–G), suggesting that CR inhibited adipogenesis through the downregulation of *Fasn*, *Plin2*, and *Slc2a4*.

### 3.5. Effects of CR on Systemic Metabolism in HFD-Induced Male Mice

**Figure 7:**
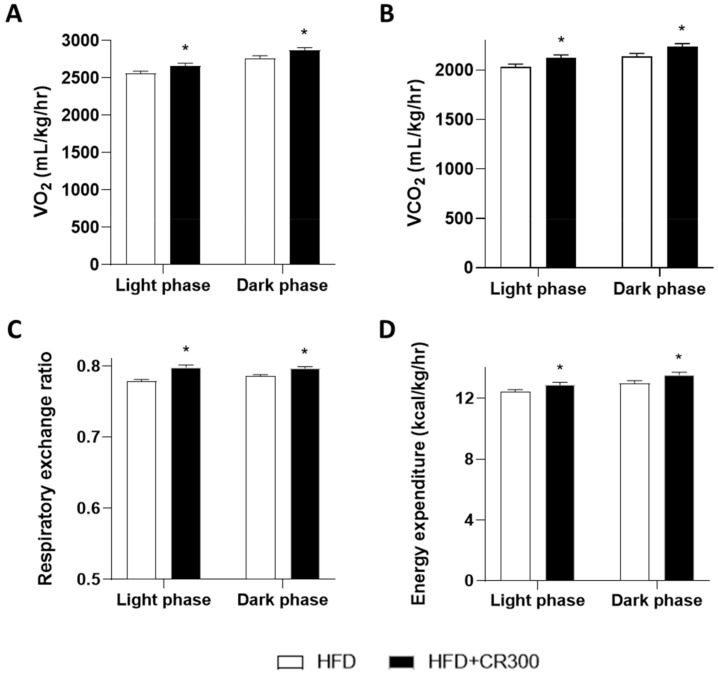
Comparison of the metabolic profiles of the HFD and HFD + CR300 groups. Mice were fed different concentrations of CR extract at 300 mg/kg/day for 12 weeks. Metabolic profiles of (**A**) average VO_2_, (**B**) average VCO_2_, (**C**) respiratory exchange ratio, and (**D**) energy expenditure were measured. VO_2_, average oxygen consumption; VCO_2_, average carbon dioxide production; ND, normal diet; HFD, high-fat diet; CR, CR extract administration; * *p* < 0.05 vs. HFD (unpaired *t*-test).

The etiology of obesity presents increased weight gain with a high risk of developing metabolic syndrome due to metabolic derangements, including impaired insulin sensitivity, glucose tolerance, dyslipidemia, high blood pressure, and abdominal obesity in the HFD rodent model [41]. Therefore, metabolic animal monitoring is a powerful tool for investigating whole-body metabolic alterations [42]. However, due to the limited metabolic cage numbers and the fact that the highest anti-obesity effect of CR against HFD-induced obesity phenotype was observed in the HFD + CR300 group, we examined the systemic metabolic parameters of the HFD + CR300 group compared to the HFD group. Mice given ND, HFD, or HFD with CR 300 mg/kg/day for 12 weeks were individually placed in metabolic chambers, and the average oxygen consumption (VO_2_), average carbon dioxide production (VCO_2_), respiratory exchange ratio, and energy expenditure were measured. HFD-induced and CR300 mice showed decreased average VO_2_, VCO_2_, respiratory exchange ratio, and energy expenditure compared to the ND group during both light and dark cycles (Supplementary Figure S7). As lean mass is principally responsible for O_2_ consumption, the amount of inspired and expired O_2_ and CO_2_ is used for the indirect measurement of heat production and subsequently calculates energy expenditure [43]. In addition, a metabolic parameter of the respiratory exchange ratio is considered an indicator of the proportion between the substrate (carbohydrate) and fuel (fat) that promotes energy production [44]. As expected, because mice are nocturnal rodents, increased VO_2_, VCO_2_, respiratory exchange ratio, and energy expenditure were observed in the dark phase (Figure 7). The administration of 300 mg/kg/day of CR extract resulted in increased average VO_2_, VCO_2_, and respiratory exchange ratio, indicating increased energy production, compared to the HFD group (Figure 7A–C). Although a high food intake or low energy expenditure influences weight gain in the HFD obese model, our results showed no difference in food intake between the HFD and HFD + CR300 groups (Supplementary Figure S8). However, a significant increase in energy expenditure was observed in the HFD + CR300 group compared to the HFD group (Figure 7D). These results suggest that CR extract administration improved HFD-induced dysregulation of energy metabolism. However, a study demonstrated limited effects of natural products on metabolic profiles, suggesting decreased body weights of obese mice probably due to changes in adipogenesis and/or lipogenesis, rather than changes in metabolic profiles [45].

### 3.6. Gender-Specific Differences in the Effects of CR in HFD-Induced Obese Mice

The prevalence of obesity in women is twice that in men [46]. Generally, females have greater fat storage than in males due to lower rates of fat oxidation and higher expression of adrenoceptors with lower adrenergic sensitivity, resulting in more subcutaneous fat [47]. A study demonstrated that the pharmacological effects against diet-induced obesity differ according to gender [48]. The authors described that diet-induced obese phenotypes between male and female mice in response to HFD were similar, but female mice were more susceptible to anti-obesity effects in terms of reducing body weight, improving glucose tolerance, and hyperinsulinemia.

Previous studies showed that CR at 75 mg/kg/day reduced weight gain in HFD-induced obese female mice [19]. However, in the present study, our results showed that treatment with 75 mg/kg/day of CR extract did not prevent weight gain in HFD-induced obese male mice, indicating that the anti-obesity effects of CR extract were more prominent in female mice than male mice. However, the overall anti-obesity effects of CR extract in both male and female mice were observed.

In addition, the magnitude of anti-obesity effects of CR150 and CR300 was not proportional to the dose, and there were no differences between CR150 and CR300 for all the data in HFD-induced obese mice. These results suggest that the minimum effective dose of CR extract for the evaluation of anti-obesity effects in animal models, regardless of gender differences, was 150 mg/kg/day. Thus, our results demonstrate the anti-obesity effects of CR extract and provide the optimal dose for preclinical studies to further investigate the tolerability and safety of CR extract administration in large animals.

## 4. Conclusions

In conclusion, we examined the anti-obesity effects of *Cornus officinalis* and *Ribes fasciculatum* extract administration in HFD-induced obese male mice. Administration of CR extract inhibited body weight gain by decreasing the expression of adipogenic inducible genes in HFD-induced obese male mice. In addition, CR treatment improved the impaired glucose metabolism, insulin action, and dysregulation of energy metabolism in HFD-induced obese male mice, resulting in a reduction in elevated biochemical obesity parameters in plasma, as well as inhibition of hepatic steatosis in the liver and adipocyte size enlargement in fat tissue. These findings suggest that CR may be suitable as a potential anti-obesity herbal medicine.

## Data Availability

The data presented in this study are available on request from the corresponding author.

## Supplementary Materials

The following are available online at https://www.mdpi.com/article/10.3390/ani11113187/s1: Figure S1. HPLC chromatograms of (A) morroniside and loganin standards, (B) *Cornus officinalis* extract, (C) 4-hydroxybenzoic acid (4-HBA) standard, and (D) *Ribes fasciculatum* extract; Figure S2. HPLC chromatograms of (A) of 4-hydroxybenzoic acid (4-HBA) and loganin standards and (B) CR extract. CR; *Cornus officinalis* and *Ribes fasciculatum* extract; Figure S3. (A) Body weight changes of mice during 12 weeks of ND provided with CR extract (300 mg/kg/day). (B) Total mouse body weight at the end of the experiment; Figure S4. Oral glucose tolerance test (OGTT) of ND mice given CR extract (300 mg/kg/day) in 12 weeks. (A) Time course of blood glucose levels during the total glucose tolerance test. (B) Representative blood glucose levels at 15 min of glucose tolerance test; Figure S5. Insulin tolerance test (ITT) of ND mice given with CR extract (300 mg/kg/day) in 12 weeks. (A) Time course of blood glucose levels during the total insulin tolerance test. (B) Representative blood glucose levels at 45 min of insulin tolerance test; Figure S6. Effects of HFD-induced obesity on plasma profiles. Plasma levels of (A) aspartate aminotransferase (AST), (B) alanine aminotransferase (ALT), (C) triglyceride, and (D) total cholesterol were examined using DRI-CHEM NX500. ND; normal diet, HFD; high-fat diet, * *p* < 0.05 vs. ND (unpaired *t*-test); Figure S7. Comparison of metabolic profiles between the ND and HFD groups. Mice were fed ND or HFD for 12 weeks. Metabolic profiles in terms of (A) average VO_2_, (B) average VCO_2_, (C) respiratory exchange ratio, and (D) energy expenditure were evaluated. VO_2_; average oxygen consumption, VCO_2_; average carbon dioxide production. ND; normal diet, HFD; high-fat diet. * *p* < 0.05 vs. ND (one-way ANOVA with Tukey’s honestly significant difference post hoc test); Figure S8. Comparison of metabolic profiles between the HFD and HFD + CR300 groups. Mice were fed HFD or HFD + CR300 for 12 weeks. Average (A) food intake and (B) drink consumption during the metabolic cage analysis were measured. HFD; high-fat diet. CR; CR extract administration; Table S1. Calibration curve parameters for 4-hydroxybenzoic acid and loganin; Table S2. Results of accuracy determination by analyzing loganin and 4-hydroxybenzoic acid at known concentrations; Table S3. Results of repeatability and intermediate precision.
