# Supplementary material for: Anti-Obesity Effects of Combined Cornus officinalis and Ribes fasciculatum Extract in High-Fat Diet-Induced Obese Male Mice
Source: Animals (Basel). 2021 Nov 8;11(11):3187. doi: 10.3390/ani11113187 (PMC8614376; doi:10.3390/ani11113187)
Supplement: Supplementary file 1 [file animals-11-03187-s001.zip › animals-1433442-supplementary.pdf]

Anti-obesity Effects of combined *Cornus officinalis* and *Ribes fasciculatum* extract in high fat diet induced male mice

Eunkuk Park[1,2,†], Chang Gun Lee [1,2], Hyoju Jeon[1,2], Hyesoo Jeong [3], Subin Yeo[3], Yoonjoong Yong[3] and Seon-Yong Jeong[1,2,*]

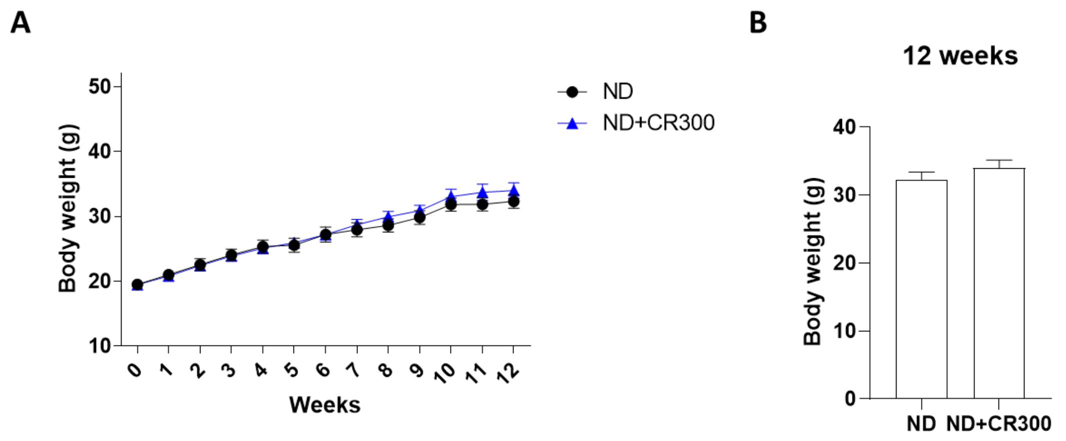

Supplementary Figure S1. (A) Body weight changes of mice during 12 weeks of ND provided with CR extract (300 mg/kg/day). (B) Total mouse body weight at the end of the experiment.

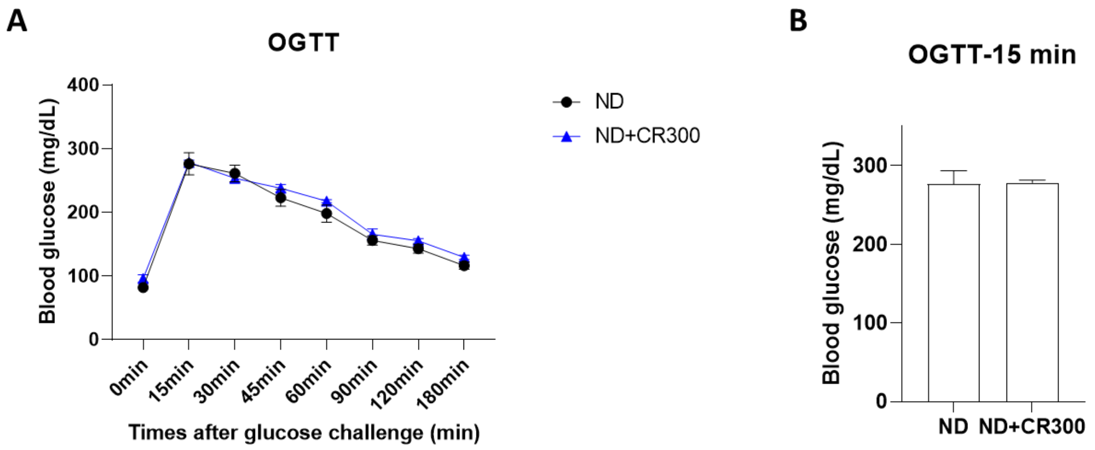

Supplementary Figure S2. Oral glucose tolerance test (OGTT) of ND mice given with CR extract (300 mg/kg/day) in 12 weeks. (A) Time course of blood glucose levels during the total glucose tolerance test. (B) Representative blood glucose levels at 15 minutes of glucose tolerance test.

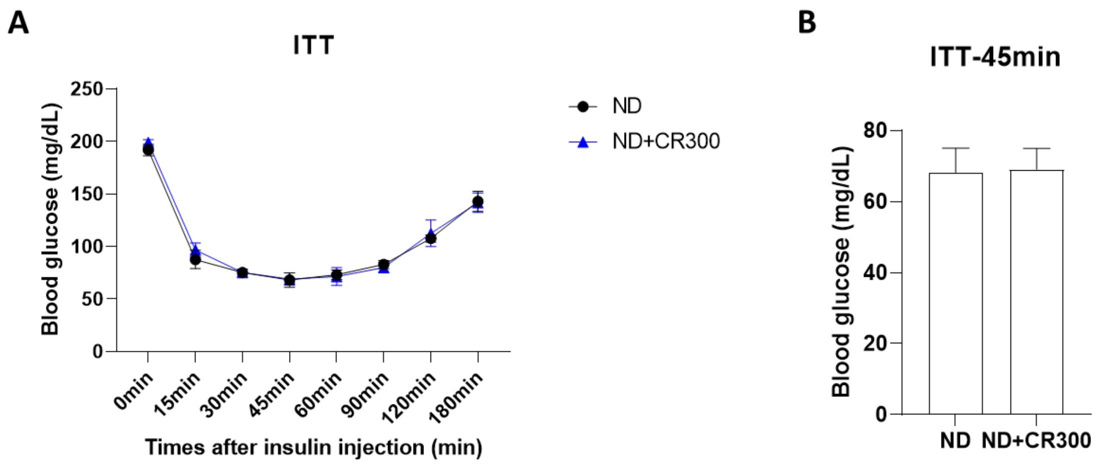

Supplementary Figure S3. Insulin tolerance test (ITT) of ND mice given with CR extract (300 mg/kg/day) in 12 weeks. (A) Time course of blood glucose levels during the total insulin tolerance test. (B) Representative blood glucose levels at 45 minutes of insulin tolerance test.

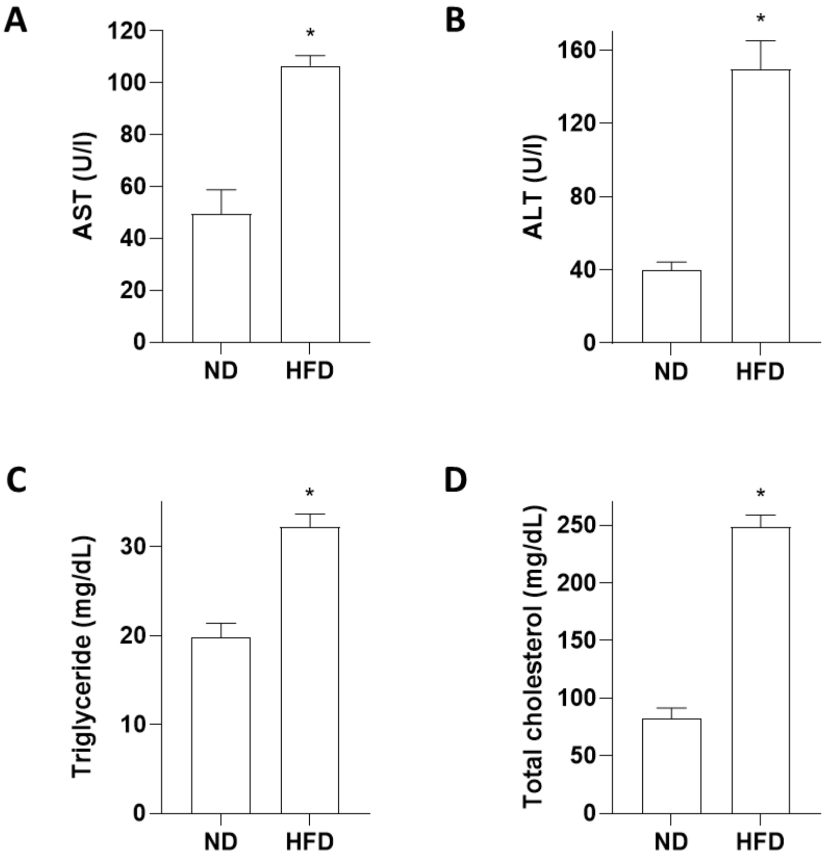

Supplementary Figure S4. The effects of HFD-induced obesity on plasma profiles. Plasma levels of (A) AST, (B) ALT, (C) triglyceride and (D) total cholesterol was examined using DRI-CHEM NX500. ND; normal diet, HFD; high-fat diet, $^{*}p < 0.05$ vs. ND (unpaired t-test).

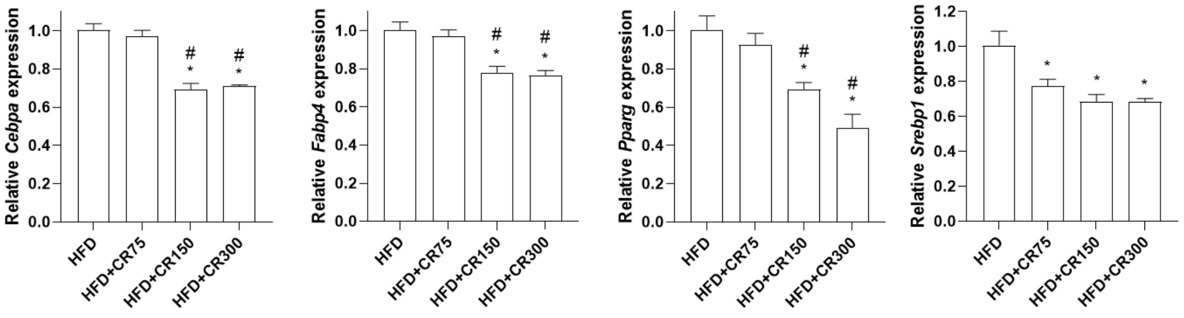

Supplementary Figure S5. The effects of CR extract administration on HFD-induced adipogenesis associated genes. mRNA expression levels of adipogenesis-associated genes in white adipose tissue were examined by qRT-PCR. Relative expression levels of *Cebpa*, *Fabp4*, *Pparg*, *Srebp1* were normalized with mouse *Gapdh* expression. HFD; high-fat diet, CR; CR extract administration, $^{*}p < 0.05$ vs. HFD $^{\#}p < 0.05$ vs. HFD+CR75 (one-way ANOVA with Tukey's honest significant difference post hoc test).

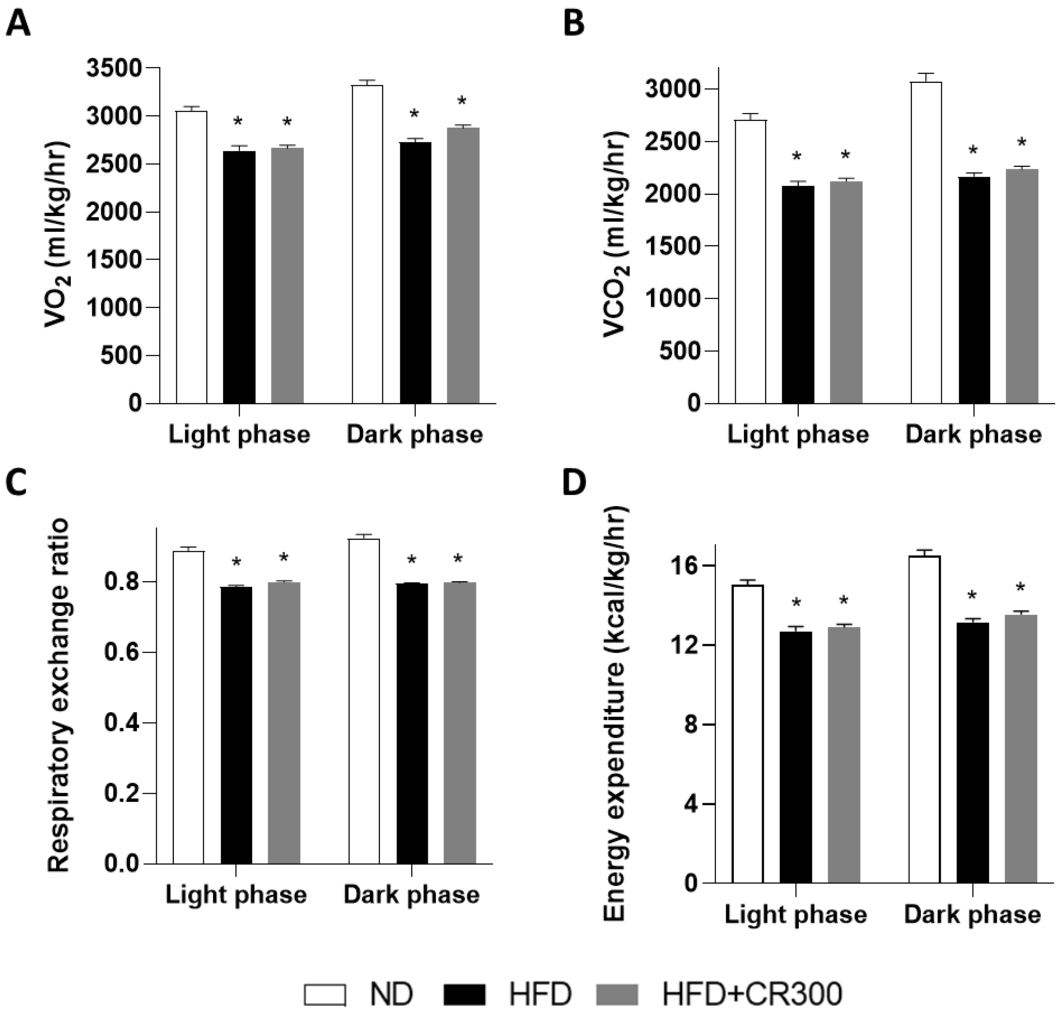

Supplementary Figure S6. In comparison with metabolic profiles between ND and HFD group. Mice were fed with ND or HFD for 12 weeks. Metabolic profiles of (A) average VO2, (B) average VCO2, (C) respiratory exchange ratio and (D) energy expenditure were measured. VO2; average oxygen consumption, VCO2; average carbon dioxide production. ND; normal diet, HFD; high-fat diet. $^{*}p < 0.05$ vs. ND (one-way ANOVA with Tukey's honest significant difference post hoc test).
